# Estimating Toxicity Putative Mechanisms from Smoking Residual Substances Using a Whole-Cell Bioreporter System

**DOI:** 10.3390/bios15110733

**Published:** 2025-11-03

**Authors:** Tal Bar, Marilou Shagan, Esti Kramarsky-Winter, Robert S. Marks, Karina Golberg, Ariel Kushmaro

**Affiliations:** 1 Avram and Stella Goldstein-Goren Department of Biotechnology Engineering, Ben-Gurion University of the Negev, Beer-Sheva 84105, Israel; bta@post.bgu.ac.il (T.B.); marilous@bgu.ac.il (M.S.); esti.winter@gmail.com (E.K.-W.); rsmarks@bgu.ac.il (R.S.M.); 2 Ilse Katz Center for Nanoscale Science, Ben-Gurion University of the Negev, Beer-Sheva 84105, Israel; 3 Department of Life Sciences, Achva Academic College, Yenon 79804, Israel; 4 School of Sustainability and Climate Change, Ben-Gurion University of the Negev, Beer-Sheva 84105, Israel

**Keywords:** bioluminescence, bacteria, bioreporter, cigarette, smoke, toxicity, oxidative stress

## Abstract

Cigarette smoking is known to be an unhealthy activity that can cause a number of human diseases, including chronic obstructive pulmonary disease (COPD) and lung cancer. It was further reported that even being exposed to secondhand cigarette smoke can affect human health. To assess the toxicity of the smoke from different cigarette brands, an artificial smoking device was developed, and three fractions designated, Filter Fraction, Smoke Fraction and Tar Fraction, were prepared from the smoke of each brand. Then, to elucidate possible effects of some of the toxins found in cigarette smoke, we investigated their effects in vitro using a bioluminescent bacterial array that comprises three bacterial strains. Using this array, we compare smoke from three cigarette brands, each with different tar and nicotine contents. GC-MS analysis showed that the cigarette smoke extracts (fractions) from different brands differed in their compositions and chemical concentrations. The results further showed that, in general, cigarette smoke triggered mainly an oxidative stress reaction in our bacterial models. The Smoke Fraction was tested for sequential smoking rounds and found to produce cumulative effects following each subsequent smoking cycle for all three cigarette brands. Finally, it was found that cigarette smoke and its specific components are toxic at various degrees with the Smoke Fraction, acting as oxidative stressors, and that this can be effectively analyzed using bioreporter panel arrays.

## 1. Introduction

Cigarette smoking has been investigated as a major cause of several respiratory pulmonary diseases, including lung cancer [1], chronic obstructive pulmonary disease (COPD) [2], and heart disease, to name but a few [3]. Globally, to date, there are about 1.1 billion cigarette smokers and billions of passive smokers, and as a result, there are reports of more than five million smoking-related deaths every year [4]. The debilitating health effects of cigarettes are due to the thousands of different chemicals they contain, including 70 different known carcinogens. Among the compounds found in cigarettes are benzenoids, aromatics, polyaromatics, alkanes, metals, and nicotine [5,6]. Each chemical family is known to cause different toxic effects that may act synergistically with other compounds, causing possible additive toxic effects [6]. When these compounds are heated and combusted, the resulting smoke contains the products and byproducts of that combustion. Upon inhalation, the smoke enters the respiratory system, where it can stick to the epithelial cells of the human airways in the form of a sticky layer of “tar” [7,8]. This tar forms a layer on the lung epithelium and can negatively affect the regular breathing process by damaging the cilia, whose function is to cleanse the lungs of inhaled debris and microbes [9]. Some of the tar along with other compounds of the smoke can also be absorbed into the bloodstream, where they increase the risk to other organs, and therefore, may cause the development of additional diseases [9].

**Figure 1:**
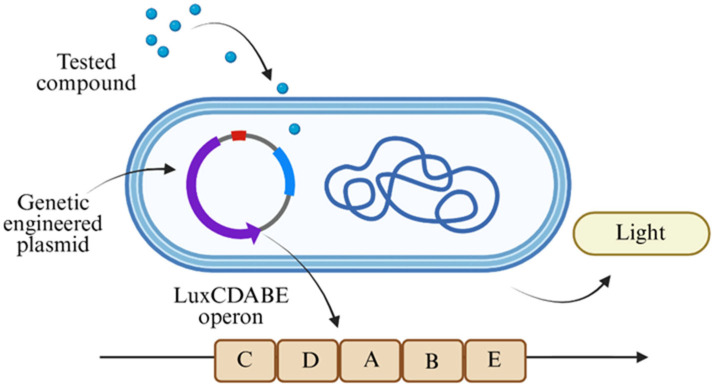
Schematic of the whole-cell bacterial bioreporter strain comprising genetic engineered plasmid with incorporated LuxCDABE operon, created using https://www.biorender.dev which accessed on 1 January 2025.

To elucidate the mechanism of action of the components of cigarette smoke, numerous toxicity tests have been developed [10], including cytotoxic assays, genotoxic assays, gene mutation assays, and in vitro micronucleus assays [11,12]. Each method measures and identifies toxicity trends based on different molecular characteristics. Several assays involve intercellular components like proteins and DNA, while in other methods, whole-cell units or even in vivo models are tested [13]. These methods, however, require intensive protocols and may be expensive to implement on large scales, dictating the need for more comprehensive and easily implementable methods. One such method is using whole-cell bacterial bioreporters, each strain of which contains a modified plasmid that has undergone genetic change (Figure 1). By exposing these bioreporters to different compounds, toxicity identification and analysis is obtained by measuring light emission intensities. A LuxCDABE operon is located under the same promotor that relates to specific repair genes, with each strain under a different promotor. While the bioreporter is exposed to a stressor compound that initiates the expression of specific proteins, the operon is simultaneously transcribed, and the luciferase enzyme produces light. A higher toxicity pattern of the tested compound is correlated to the light intensity, and by measuring this the toxicity patterns can be identified [14]. Easy to use, the whole-cell biosensor tool is also relatively cheap, and it produces results rapidly compared to the longer time scales required when working with the aforementioned toxicity methods [15]. Indeed, bioreporter analyses are used in different fields, like environmental monitoring, food industry, and in medical examinations [15]. Additionally, using this method allows for a variety of different components to be screened and evaluated simultaneously and with multiple replicates to ensure that more accurate results are obtained. This method can be used to directly identify the specific bioavailable toxins among the different components of cigarette smoke. In this study, a bioluminescent bacterial array was used to monitor the toxicity of cigarette smoke fractions.

## 2. Materials and Methods

### 2.1. Materials

All materials were purchased at Sigma-Aldrich (Rehovot, Israel): nicotine (CAS No. 54-11-5), alkane components, including tetradecane (CAS No. 629-59-4), heptadecane (CAS No. BCBX3899), heptacosane (CAS No. 593-49-7), octadecane (CAS No. 593-45-3), dodecane (CAS No. 112-40-3), eicosane (CAS No. 112-95-8), pentadecane (CAS No. 629-62-9), ring-shape components, including glyceryl triacetate (triacetin) (CAS No. 102-76-1), 2,4 di-tert butylphenol (CAS No. BCCB3522), methyl stearate (stearic acid) (CAS No. 112-61-8), PAH components, including benz[a]anthracene (CAS No. 56-55-3), benzo[b]fluoranthene (CAS No. 205-99-2), benzo[a]pyrene (CAS No. 50-32-8), chloroform (CAS No. 67-66-3), ampicillin (CAS No. 69-52-3) and positive control materials, including mitomycin C (EC No. 200-008-6), ethanol (LOT No. 19-009101-78), hydrogen peroxide (LOT No. A0325692), acetonitrile (LOT No. 1083059), Difco Luria–Bertani (LB) broth, Miller (10 g L^−1^ tryptone; 5 g L^−1^ yeast extract; 10 g L^−1^ NaCl) and Difco LB agar, Miller (10 g L^−1^ tryptone; 5 g L^−1^ yeast extract; 10 g L^−1^ NaCl; 15 g L^−1^ agar), and TWEEN 80 (CAS No. 9005-65-6).

Cigarette brands were distinguished from one another based on their respective tar and nicotine contents, which were both classified as low, medium, or high.

### 2.2. Bacterial Strains

Three modified *Escherichia coli* strains (TV1061, DPD2794 and DPD2511) supplied by S. Belkin (Hebrew University, Jerusalem, Israel) were used in this study (Table 1). Each strain comprised a genetically engineered plasmid that was distinguished by the location of the inserted reporter operon. A LuxCDABE operon was inserted into each plasmid next to the specific genes for which we tested, and the genetically altered bacterium produced luminosity when exposed to a specific stressor [16]. For each strain, the operon was located under a different promoter: heat-shock cytotoxic stress grpE, SOS genotoxic stress recA, and oxidative stress katG promoters for TV1061, DPD2794 and DPD2511, respectively [16,17,18].

### 2.3. Growth Conditions

To culture the strains and perform the luminescence tests, 10 mL of LB was added to a sterile 50 mL polypropylene tube supplemented with 10 µL of 100 µg/mL ampicillin dissolved in double distilled water (DDW). After adding the antibiotic, several colonies of a single bacterial strain that were grown overnight in a Petri dish at 37 °C were inserted into the tube. The bacterial solution was then incubated overnight at 140 rpm and 37 °C (Table 1). The next day, 200 µL of this solution was transferred to 20 mL of fresh LB in another sterile 50 mL tube and incubated for another 2 h at 30 °C without shaking. These preparatory steps were needed for the bacteria to reach the early exponential phase, which was determined by using a spectrophotometer (Amersham Bioscience, Piscataway, NJ, USA) to measure the solution’s optical density (OD), which was about 0.2.

### 2.4. Bioluminescence Assay

The bioluminescence of all three strains was measured at 490 nm and 30 °C for 24 h at 5 min intervals with continuous shaking in a white, opaque 96-well microtiter plate. Each well contained 10 µL of different concentrations of tested sample or control (TV1061–ethanol 4% (*v*/*v*), DPD2794–mitomycin C 80 ppb, and DPD2511–H_2_O_2_ 0.035 mg/mL for positive control and LB for negative control), and 90 µL of bacterial culture. To test the hydrophobic smoke fractions, 1% (*v*/*v*) TWEEN 80 was used as a mediator between the culture, and the hydrophobic fraction was tested. In every measurement in the luminometer, agitation was performed in order to reach homogenous conditions in each well.

### 2.5. Cigarette Smoke Fractions

**Figure 2:**
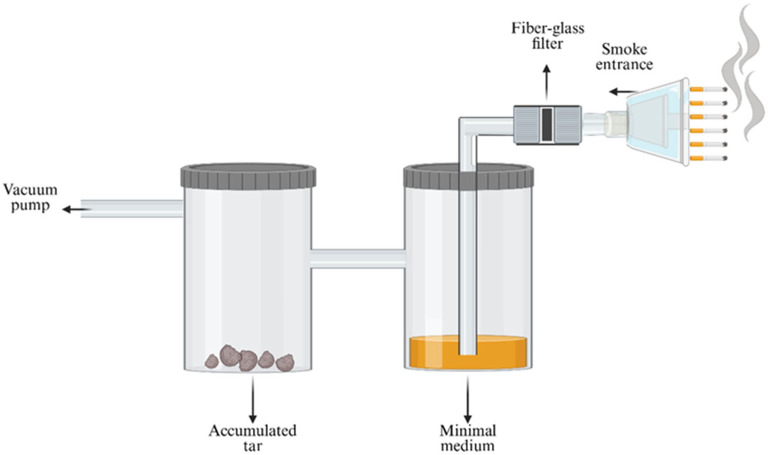
Schematic of the artificial smoking device from which three different fractions were obtained: Filter Fraction with components that adsorbed to the wet glass filter, Smoke Medium with components that passed through a dry glass filter and then dissolved in the minimal medium in the first flask, and Tar Fraction, which contained the compounds that did not dissolve in the minimal medium, but instead flowed into the second flask, where they accumulated as tar. Using a vacuum pump, the apparatus could “smoke” six cigarettes during each smoking cycle. Created using https://www.biorender.dev which accessed on 1 January 2025.

For the toxicity bioassays, three different cigarette brands were selected based on their tar and nicotine contents. The nicotine and tar yields were 1.12 ± 0.03 mg/cig and 18.3 ± 0.5 mg/cig, 1.4 ± 0.02 mg/cig and 22.9 ± 0. 6 mg/cig and 1.72 ± 0.1 mg/cig and 24.9 ± 0.7 mg/cig for the brands with low, medium, and high nicotine and tar contents, respectively [19]. The components of the cigarette smoke were extracted into three different solutions using the artificial smoking device, and they were named Filter Fraction, Smoke Medium, and Tar Fraction (Figure 2). The artificial smoking device could “smoke” six cigarettes every single smoking cycle, and a total of ten smoking cycles were performed, which in total included 60 cigarettes. The smoke flowed into the smoking system through a 1 µm fiberglass filter (Whattman, Sigma-Aldrich (Rehovot, Israel)) with a diameter of 55 mm, which was replaced after every smoking cycle. Two extraction pathways were tested. In the first, the filter was wet, and most of the smoke was trapped on it. The substances trapped in the filter were extracted by using 20 mL of chloroform, and the resulting solution was named the Filter Fraction. In the second pathway, a dry filter was used, through which most of the smoke passed and then entered the first and second flasks (Figure 2). The first flask, which contained FAB minimal media without any carbon source (MgCl_2_∙6H_2_O, NaCl, KH_2_PO_4_, Na_2_HPO_4_, (NH_4_)_2_SO_4_, CaCl_2_ purchased at Sigma-Aldrich (Rehovot, Israel)), trapped mainly the hydrophilic components of the cigarette smoke in the medium. The extract from the first flask was named the Smoke Medium. The compounds that were not trapped in the medium of the first flask continued to the second flask where they accumulated as deposited tar. The resultant tar accumulation was extracted after two cycles, which correlated to twelve cigarettes that were “smoked” with 10 mL ethanol and 5 mL chloroform, and named the Tar Fraction. Each fraction was tested and analyzed for its toxicity.

### 2.6. Cumulative Test

A total of ten smoking cycles were performed. The Smoke Medium solution in the first flask was tested for the cumulative effect of each subsequent smoking cycle. After every smoking cycle, 10 mL of the minimal medium solution with the dissolved smoking components was removed for further testing and the filter was replaced by a new dry filter for the next cycle. In total, 10 sequential samples were collected.

### 2.7. Gas Chromatography–Mass Spectrometry Analysis

To analyze the sample compositions, a gas chromatography–mass spectroscopy (GC-MS) (Agilent Technologies, Santa Clara, CA, USA) analysis was performed. The unit’s Agilent 7890B GC was connected to an Agilent 5977A single-quadrupole mass-selective detector. The instrument is equipped with a 100-vial autosampler, an NIST02 MS, and an ACD Labs MS Manager (B.07.02 version) software package (Agilent Technologies, Santa Clara, CA, USA) for mass spectra interpretation and structure elucidation). The column type was 35% phenyl methyl siloxane for MS; length 30 m; 0.25 mm, I.D. and 0.25 µm film thickness; temperature was programmed at 50 °C for 5 min to 20 °C at 3 °C min^−1^ to 320 °C at 3 °C min^−1^. Transfer line temperature 280 °C and total run time is 21.7 min. A carrier (helium) gas flow rate of 2 mL min^−1^ was applied. The vapor-injection volume of 1 µL with split of 1 to 10 into the gas chromatography.

### 2.8. TOC Analysis

Samples from the Smoke Medium fractions were prepared for total organic carbon (TOC) analysis to measure the average carbon content of a single smoking cycle. Two ml of the Smoke Medium solution was filtered through a MILLEX GP 0.22 µm filter (Sigma-Aldrich (Rehovot, Israel)). To obtain more accurate results, the solutions were diluted according to their cumulative effect resulting from the cycle number (the first cycles were diluted by 10, the middle and last cycles were diluted by 20 and 50, respectively, due to their higher carbon contents). These samples were heated to 680 °C and inserted into the TOC-L series analyzer (Shimadzu, Riverwood Drive, Columbia, MD, USA) at a gas flow of 150 mL/min. The calibration solution was DDW.

### 2.9. Data Analysis

The bioluminescence signal results are presented as induction factor (IF) values. The IF values were calculated using the following Formula (1):

(1) $$IF=\frac{MIF}{CIF}$$

where MIF (material induction factor) represents the maximum bioluminescent signal of a tested sample and CIF (control induction factor) represents the maximum bioluminescence signal of the negative control [20]. IF values greater than 1.5 indicated oxidative stress, and thus, they were accompanied by reporter gene expression, while IF values less than 0.6 indicated general toxicity and the inhibition of cell growth. IF values between 0.6 and 1.5 were designated as no or low toxic effect. T-test, one-way ANOVA, and two-way ANOVA statistical tests were performed.

## 3. Results and Discussion

This study evaluated the types and levels of toxicity found in cigarettes by using a bacterial bioreporter panel that included three different toxicity reporter strains. The genetically modified bacteria bioreporters each emit different light when they are exposed to different toxins. The use of this bioluminescent bacterial array enabled screening of the components of cigarette smoke to determine their toxicity. The toxicity trends of specific mechanisms were obtained according to the specificity of the bioreporter. The bioreporter-based method is rapid and its results are reliable, rendering it an efficient test with which to understand and identify the toxicity of single components or mixtures of several compounds. The three bioreporters used in this study were *E. coli* strains (TV1061, DPD2794 and DPD2511), whose luminescence is indicative of cytotoxicity, genotoxicity and oxidative stress, respectively. For the toxicity analysis, hydrophobic materials were dissolved in chloroform and supplemented with TWEEN 80, which was also tested for oxidative stress activity (Figure S1). The IF value that was obtained for TWEEN 80 was close to 1 and for chloroform + TWEEN 80 was around 2.5. Thus, in comparison to the fractions, IF values were found to be significantly higher than the pure chloroform. Furthermore, based on previous research that found no high oxidative stress effect of chloroform to the DPD2511 [21] it was decided to use LB as the negative control.

### 3.1. GC-MS Analysis of Cigarette Composition

**Figure 3:**
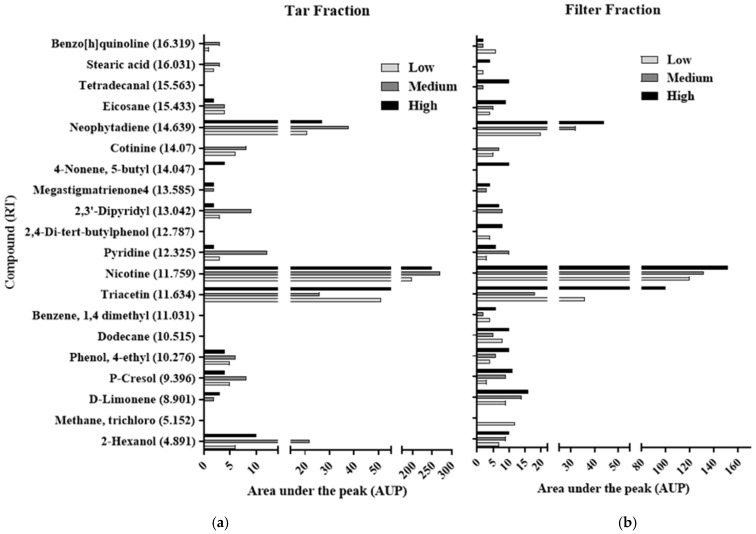
GC-MS analysis of the Tar Fractions and the Filter Fractions from the three cigarette brands. The y-axis lists the components, from the smallest at the bottom with a smaller retention time to the largest at the top of the axis with higher retention time, and the x-axis, which represents the area under the peak (AUP), correlates to concentration. (**a**) GC-MS analysis of Tar Fraction for three cigarette brands that contain high, medium, and low tar and nicotine contents. (**b**) GC-MS analysis of the Filter Fraction for three cigarette brands that contain high, medium, and low tar and nicotine contents.

In this study, three cigarette brands that differed in their tar and nicotine contents—a low content brand (LCB), a medium content brand (MCB), and a high content brand (HCB)— were tested for their toxicity reaction. A GC-MS analysis was used to identify the material compositions of the brands and the differences between them (Figure 3). The results of the GC-MS analysis indicate that the cigarette brands differed in terms of their compositions and the chemical characteristics of the compounds found in each. Several compounds, including nicotine [22], triacetin [23], and stearic acid [24], were identified in the fractions from all three of the cigarette brands in agreement with previous findings. Compositional differences between the fractions of the three brands were identified (Figure 3a,b). The Filter Fraction was obtained using a wet filter, which absorbed most of the smoke that was pumped from the cigarettes and contained the combusted materials, while the Tar Fraction contained smoke components that were neither absorbed to the dry filter nor dissolved in the aqueous minimal media in the first flask (Figure 2).

The GC-MS analysis showed that the fractions obtained from the wet and dry filter setups (the Filter Fraction and the Tar Fraction, respectively) differed in terms of composition and concentration. Significant overlap was seen between the two fractions, but there were several differences. Trichloro methane, with its retention time (RT) of 5.152, was found only in the HCB, and only in the Filter Fraction which contained six components that were absent from its Tar Fraction, including stearic acid, dodecane and 2,4 di-tert-butyl phenol, which were tested separately. A similar pattern was observed for the other two brands tested in this study. For the LCB, 16 compounds were found in the Filter Fraction, while the Tar Fraction contained 12 compounds, and for the MCB, 16 compounds were found in its Filter Fraction and 13 were found in its Tar Fraction (the differences shown are for the most abundant compounds that were determined by the GC-MS).

The observed differences between the brands in the numbers of compounds that were found in their Filter Fractions vs. in their Tar Fractions can be explained by the fact that the wet filter may have absorbed more types of molecules, both solid and gaseous, compared to the tar phase that accumulated in the second flask and that was comprised mainly of extracted solids with a lower variety of compounds [25]. The quantity of nicotine also differed between the two fractions. The quantities of nicotine found in the Tar Fraction vs. in the Filter Fraction were about 64% higher in the HCB and LCB cigarettes and about 103% higher in the MCB cigarettes. The higher concentrations observed in the Tar Fraction may be due to the hydrophilic characteristics of the nicotine, which was removed from the Tar Fraction using ethanol (as described in the Materials and Methods), while it was removed from the Filter Fraction using chloroform as the solvent. Likewise, although cigarette filters reduce the number of components that enter the lungs due to the absorption of those components by the filter, the typical cigarette filter is an insufficient barrier that allows some of the components of the cigarette smoke to pass through and accumulate on the lung tissue, from where they can even pass into the bloodstream [26]. Two other components that were found in relatively high abundance compared to the other components in both the Filter and the Tar Fractions were neophytadiene and triacetin. While neophytadiene is a major constituent of the essential oils in tobacco leaves [27], triacetin is known as an unstable molecule that degrades rapidly after its release to the air, thus producing hydroxyl radicals, which are known to be oxidative stressors [28]. This supports the results that were obtained by Watts et al. [29].

### 3.2. Toxicity Determination and Monitoring

**Figure 4:**
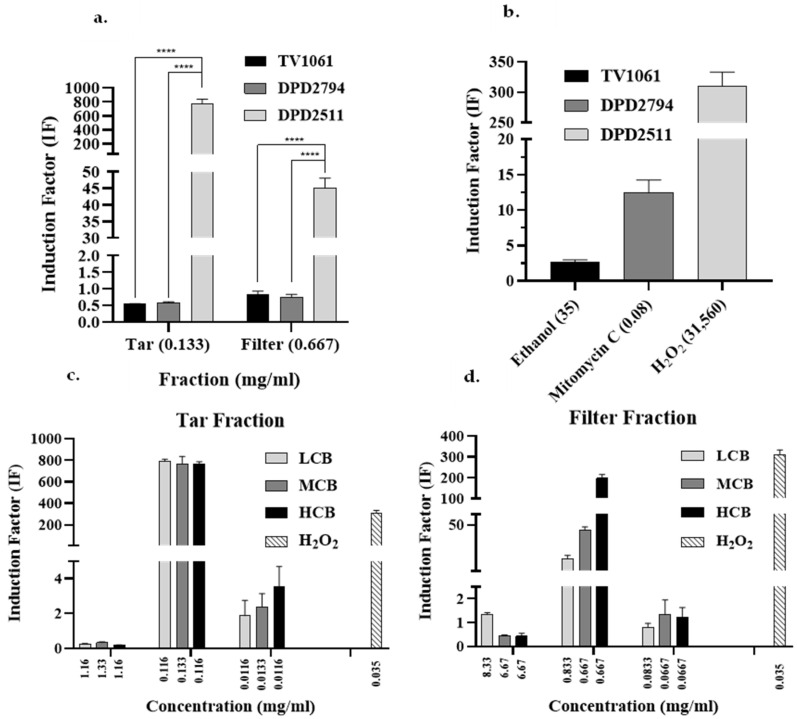
Toxicity assessment using a panel of three bioreporters and toxicity analysis of two fractions, Filter Fraction and Tar Fraction, from the smoke of three cigarette brands (LCB, MCB, and HCB) using the DPD2511 bioluminescent strain. (**a**) Toxicity analysis using DPD2794, TV1061, and DPD2511 for both the Tar Fraction and the Filter Fraction. (**b**) Positive controls including ethanol, mitomycin C, and hydrogen peroxide were tested by TV1061, DPD2794, and DPD2511, respectively, and substantial light emission above an IF of at least 2.5 were observed, indicating the activity and viability of the bioreporters. (**c**) Oxidative stress responses of the Filter Fraction testing three incremental concentrations. (**d**) Oxidative stress responses of Tar Fraction testing three incremental concentrations. For all of these solutions, the luminescence of DPD2511 was used as an indication of oxidative stress. This enabled the determination of which compound and its specific concentration triggered oxidative stress according to the IF values. In panel a, a two-way ANOVA was performed with *p* < 0.0001 **** and in panels b and c, multiple t tests were performed with *p* < 0.0001 ****.

To identify the type(s) of toxic effect generated by cigarette smoke, the Tar and Filter fractions were tested using all three bioreporters (Figure 4a). The luminescence of each bioreporter is indicative of a specific stress mechanism (Table 1). For both fractions, only the DPD2511 bacterial strain showed IF values greater than 1.5, indicating oxidative stress. The values obtained with the other two bioreporter strains were between 0.6 and 1.5, indicating low cytotoxic or genotoxic effects. In this study, therefore, the DPD2511 bioreporter was used for the remainder of the analyses. The exposure of this specific strain to our positive control oxidative stressors, H_2_O_2,_ was highest as it activated the expression of oxyR, which in turn triggers the expression of catalase, which converts the hydrogen peroxide into water and oxygen [30,31].

Optical density (OD) was measured for DPD2511 exposed to both the Tar and Filter fractions, which indicated how the bacteria reacted to the exposure via oxidative stress (Figure S2a). For both fractions, the optical densities were lower than for the negative controls, a finding that may be related to the fact that, in triggering oxidative stress in the bacteria, these fractions may have actually reduced the bacterial growth rate [32]. Though our results, which were confirmed by testing positive controls for all three strains (Figure 4b) and their performances indicating activity, only the DPD2511 bioreporter was further tested against different concentrations of the Filter Fraction, Tar Fraction and Smoke Fraction. As previously explained, each fraction was prepared using different processes and conditions, and therefore, each fraction may have contained different components in varied concentrations. Despite this, it was possible to compare the same fraction between brands. A comparison of the Filter and Tar fractions of the three cigarette brands using the DPD2511 bioreporter strain (Figure 4c,d, respectively) showed that, at the highest concentration for both fractions in all three of the cigarette brands, (except for LCB’s Filter Fraction), they are all very toxic to the bacteria. The IF values were found to be below 0.6, which indicates a strong inhibition effect on bacterial growth [20]. Moreover, the second most concentrated Tar Fraction from all three brands registered higher IF values than the Filter Fraction at the same dilution, indicating that the Tar Fraction was the most toxic. For example, the Tar Fraction of the LCB had an IF value that was almost 46 times that of the LCB’s Filter Fraction. For the MCB, the Tar fraction’s IF value was approximately 17 times that of the Filter Fraction, and for the HCB, it was four times more. It is also clear that while the IF values of the Tar Fraction are similar among the three cigarette brands, the IF values of the Filter Fraction varied between 16.88 for the LCB to 196.6 for the HCB. Thus, different compositions and concentrations were identified in both the Tar and the Filter fractions, explaining the differences in IF values.

### 3.3. Analysis of Smoke Components Using the DPD2511 Bioreporter

**Figure 5:**
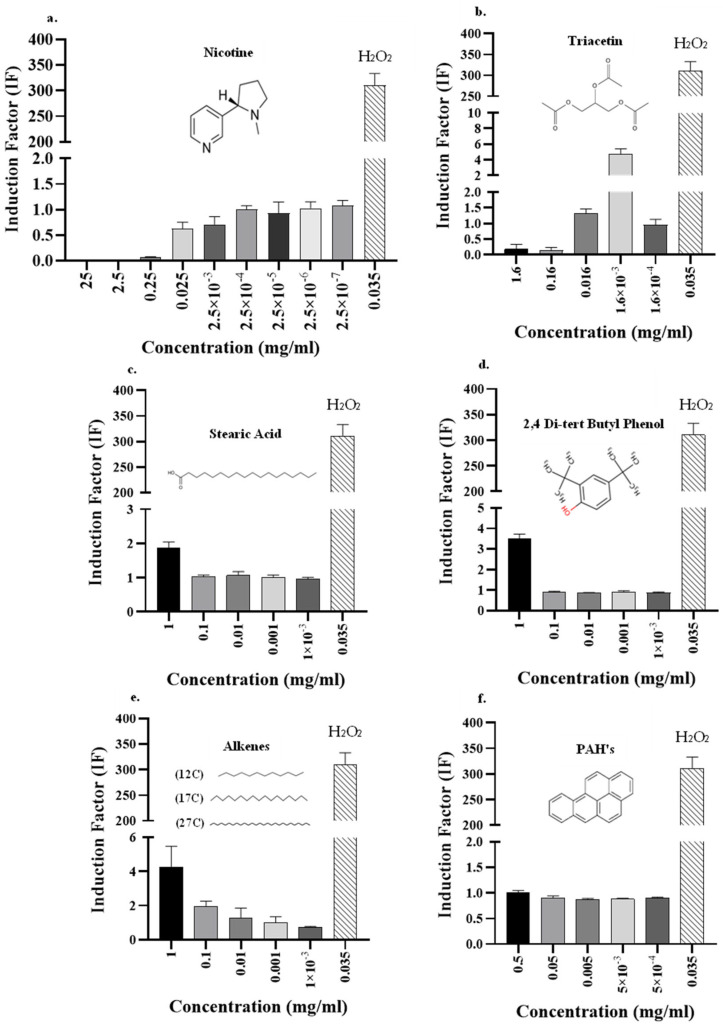
Toxicity and kinetic analyses of known isolated compounds and mixtures of compounds from cigarette smoke using the DPD2511 strain. Effects of gradually increased concentrations were tested using single compounds: (**a**) Nicotine, (**b**) triacetin, (**c**) stearic acid, (**d**) 2,4 di-tert butyl phenol, and two mixtures of (**e**) alkanes and (**f**) PAHs (poly-aromatic hydrocarbons). For all these components, the DPD2511 bioreporter for oxidative stress was used, which enabled the specific compound and its concentration to be determined, and which triggered oxidative stress according to the IF values. As a positive control, H_2_O_2_ 0.035 (mg/mL) was used.

As in previous studies [15,33], this report shows that cigarette smoking harms the smoker’s health and causes oxidative stress in the smoker’s tissues [34]. To obtain a better understanding of the source of the toxicity, two families of chemicals were tested, alkanes and poly-aromatic hydrocarbons (PAHs), which are two of the components of the particulate phase of cigarette smoke [35] (Figure 5). Additionally, a few toxic compounds that are found in cigarette tobacco were tested separately, including nicotine, stearic acid, triacetin, and 2,4 di-tert butyl phenol. In contrast to the results of Crowley-Weber et al., pure nicotine (Figure 5a) did not trigger an oxidative stress response [36]. This may be because the samples that were tested were mixtures of compounds that were extracted from cigarette tobacco, and therefore, they may have reacted differently than if they had been in their pure forms [37]. Moreover, the fractions were tested after the tobacco combustion process. As nicotine is a component of tobacco, it is possible that it underwent chemical changes during the smoking process, or it may have even been degraded into smaller molecules like pyridine, which was detected by the GC-MS analysis (Figure 3). The by-products, especially pyridine, may further contribute to the high oxidative stress that was reported by Falin et al. [38], and should be further studied in future experiments. An alternative assumption is that nicotine does not initiate oxidative stress in this specific mechanism.

Another explanation for the low IF values that were obtained in this study may be that high toxicity resulting from the high concentrations of nicotine perhaps interfered with the bacterial growth kinetics that were analyzed (Figure S2b). While solutions with lower concentrations of nicotine had growth patterns similar to that of the negative control, the three solutions with the highest concentrations presented lower growth values. A similar growth effect has been observed previously [39,40]. Figure S2b shows that for the two highest concentrations, 25 and 2.5 mg/mL, the patterns of the curves indicate bacterial death, while for the more highly diluted fraction (0.25 mg/mL), the bioluminescent pattern more closely resembles that reported for the inhibition of cell growth rather than cell death. Further dilution did not result in growth inhibition (Figure S2b). A similar pattern of high toxicity was also evident for the highest concentrations of triacetin (Figure 5b), a compound known to be unstable and that is degraded to hydroxyl radicals that lead to oxidative stress [28,29,41]. Since the IF values here were close to 0, this may indicate that triacetin may have led to bacterial growth inhibition or even bacterial cell death (Figure S2c). It can be noted that for the 1.6 mg/mL of triacetin the trend stays low, indicating bacterial inhibition or death, and for the 0.16 mg/mL dilution the trendline indicates cell growth. It is possible, therefore, that lower OD values may be a reflection of the inhibition effect of triacetin on the bioreporter, even at low concentrations. In addition, after three serial dilutions, the fourth dilution produced an IF value that was close to 5 (Figure 5b), representing gene induction and indicating that triacetin did trigger oxidative stress.

To strengthen the premise that our bioreporters can determine toxicity via oxidative stress, additional molecules were tested. For example, stearic acid (Figure 5c), a fatty acid with a long hydrocarbon chain, was also evaluated using our bioreporters [42]. In detail, 1 mg/mL of pure stearic acid produced an IF value that was indicative of oxidative stress. The same oxidative pattern was observed at 1 mg/mL for 2,4 di-tert butyl phenol (Figure 5d). For both compounds, no published studies have explicitly examined their oxidative stress effects. As can be seen in Figure 5c,d of this study, both compounds contain a hydroxyl group, which was proposed by Watts et al. to be a reactive group that can lead to oxidative stress [29].

Additional chemical families found in cigarette smoke were tested in this study (Figure 5e). The alkanes triggered induction at both concentrations examined (1 mg/mL and 0.1 mg/mL), with IF values around 4 and 2, respectively. Interestingly, the PAHs, which were previously reported by Hanzalova et al. to be oxidative stressors, did not induce an oxidative stress pattern in the present study (Figure 5f) [43]. Kim et al. found that the PAH benzo[a]pyrene, which was also examined in this research, did not trigger the katG gene, which is related to catalase–peroxidase enzyme activity and was tested in this research [44]. This finding may be explained by the behavior of PAH molecules while in the human body, where they enter the bloodstream and are exposed to enzymes that metabolize them. In our study though, bacterial bio-reporters were used, and the components were tested in their native shape. Meanwhile, in the human body, these molecules turn into products that are able to enter redox cycles, where they increase the formation of reactive oxygen species (ROS), thus causing oxidative stress [43]. Threshold concentration differences may be another reason for the induction obtained when compared with the results published by Velali, E. et al. [45], who concluded that PAHs indeed trigger oxidative stress. Jeng et al. also found that the PAH class of chemicals triggers oxidative stress, but they suggested that further study is needed to elucidate the mechanisms that are relevant to these compounds [46]. Another reason for the divergence of our results from those of previous studies is that the PAH compounds in this study were tested using only three standard PAH chemicals (benz[a]anthracene, benzo[b]fluoranthene, and benzo[a]pyrene), while in cigarettes, there is generally a mixture of many types of PAH molecules that may be acting synergistically. It is therefore likely that the combination of different compounds and their combustion products may have led to the increase in ROS [35,47].

**Figure 6:**
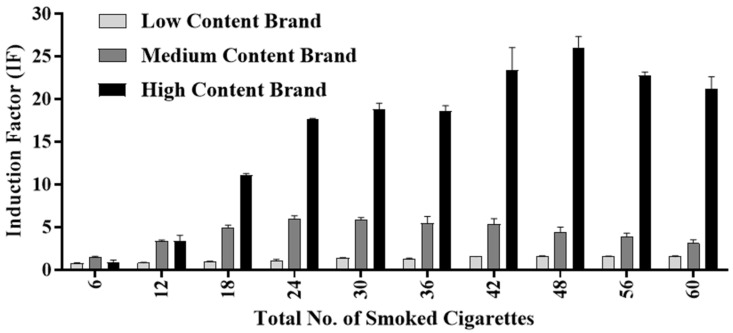
Cumulative toxicity analysis of the three cigarette brands (LCB, MCB, and HCB) using the DPD2511 strain. Six cigarettes were smoked for each cycle for a total of 60 cigarettes. Cumulative oxidative stress was tested after each smoking cycle using the DPD2511 bioreporter.

Unlike the Tar Fraction and Filter Fraction, which were diluted and tested in incremental concentrations, the Smoke Medium Fraction was allowed to accumulate for the entirety of the ten smoking cycles, and the cumulative pattern was tested (Figure 6). In each smoking cycle, six cigarettes were smoked by the artificial smoking device for a total of 60 cigarettes. To obtain a series of samples that would be representative of the human smoking habit and that would mimic an actual smoking routine, at the conclusion of each cycle, a 10 mL sample was removed from the Smoke Medium solution. The resultant ten samples were then tested, and the results were compared. This type of analysis enables a more comprehensive understanding with regard to cigarette smoking.

For both the MCB and the HCB, the oxidative stress was observed to increase, and the maximal IF values obtained due to the accumulation of Smoke Fractions were 5.97 and 26.03, respectively. The maximal IF value for the MCB was obtained after smoking 24 cigarettes, while for the HCB it was obtained after smoking 48 cigarettes, a difference that can be explained by the different compositions and concentrations of the tobacco components in the two brands [48] (Figure 3). Another result that was noticeable in both brands was the subsequent decrease in IF values that occurred after reaching these peaks, which concluded that the observed patterns of IF values were due to the accumulation of toxic components in critical concentrations, which in turn caused an inhibition of bacterial cell growth and even cell death [5,49]. Once this toxin threshold was exceeded, therefore, the bioreporter may have been inactivated or inhibited [50].

The nicotine content in the Tar Fraction was higher in the MCB than in the HCB. This may help explain the earlier peak observed in the IF value of the MCB compared to that of the HCB. It was previously reported that cigarette smoke, including tar, accumulates in the lungs and that it probably has a strong oxidative toxic effect on the smoker [51]. For the LCB, an IF value exceeding 1.5 was obtained after smoking 48 cigarettes, and the value remained between 1.5 and 1.6 for the next two cycles, representing an additional 12 cigarettes that were smoked. These findings indicate that this brand induces a lower oxidative stress effect than the other two brands, at least in the short term. This was specifically found in the fraction that was comprised mostly of compounds that are soluble in an aqueous solution. These results emphasize the generally higher toxicities of the other two brands, whose IF values were 4 and 16 times greater. Regarding nicotine specifically, an oxidative stressor [36], luminosity levels were correlated to the contents of each brand. The HCB showed the higher IF values, while the LCB showed the lowest among Smoke Medium fractions.

## 4. Conclusions

For over 60 years, cigarette smoking has been known to be a major cause of many human diseases. Cigarette smoke is a mixture of a variety of components including more than 7000 chemicals, including carcinogens and many other toxic materials, all of which directly affect the health of smokers, as well as others via secondhand smoke. In this study, three specific toxicity mechanisms were tested. Additionally, we developed tests using the DPD2511 bioreporter bioluminescent bacteria for oxidative stress by activating the oxyR mechanism. By testing different compounds separately and as mixtures and by using three different fraction phases, stress and oxidative stress patterns were reported alongside kinetic analyses. These tests indicated that at high concentrations, these toxins result in oxidative stress and could lead to the inhibition of bacterial growth. We found that there were differences between the chemical compositions of the Tar Fraction and the Filter Fraction in the smoke of the same cigarette. Similarly, the different cigarette brands, which contain different nicotine and tar contents, showed different oxidative stress induction signals. Moreover, most of the components of the fractions were shown to induce oxidative stress. We further show that at high concentrations, nicotine and triacetin could be toxic to the bioreporter bacteria and interfere with their growth, resulting in negative results. Our artificial smoking device was found to be useful for a synthetic media assembly where the carbon source came from different combustion reactions. Furthermore, we show that using bacteria bioreporters as toxicity monitors could provide a quick method of measuring toxicity patterns. However, it should be noted that as these bioreporters are genetically engineered bacteria, they react differently from mammalian cells which have different regulation pathways and thus corresponding responses to smoke toxicity therefore might be different from the bioreporter bacteria in this study and should be further investigated in future experiments. This study shows that toxicity analyses and efficient monitoring tools such as the whole-cell bioreporter provide sensitive, rapid and reproducible solution.

## Figures and Tables

**Table 1:** Strain characteristics, growth conditions, and genetic description of the three *E. coli* strains TV1061, DPD2794, and DPD2511, including plasmid designation, antibiotic name and concentration, growth temperature, and stress sensitivity.

Bacteria		Antibiotic		Temperature		
Strain	Plasmid	Name	Concentration (µg/mL)	Growth (C^0^)	Refreshment (C^0^)	Stress Sensitivity
DPD2794	pGrpELux3	Ampicillin	100	37	30	SOS
TV1061	pRecALux3	Ampicillin	100	37	30	Heat Shock (general stress)
DPD2511	pKatGLux2	Ampicillin	100	37	30	Oxidative (peroxides)

## Data Availability

Data are contained within the article or supplementary material, and further inquiries in terms of data can be found in our records in footprints (https://footprints-b291f.web.app/, accessed on 17 September 2025); authorization for access may be granted by the author (rsmarks@bgu.ac.il).

## Supplementary Materials

The following supporting information can be downloaded at https://www.mdpi.com/article/10.3390/bios15110733/s1, Figure S1: Hydrophobics solutions’ additional compounds as oxidative stressors using DPD2511 bioreporter; Figure S2: Kinetic analysis of the DPD2511 strain while exposed to cigarette fractions and single compounds. (a) Kinetic analysis of DPD2511 for both the Tar and Filter fractions from the medium content brand (MCB). (b) Kinetic analysis of DPD2511 for serial dilution concentrations of nicotine. (c) Kinetic analysis of DPD2511 for serial dilution concentrations of triacetin.

## Abbreviations

The following abbreviations are used in this manuscript:

COPD	Chronic obstructive pulmonary disease
H_2_O_2_	Hydrogen peroxide
LB	Luria–Bertani
DDW	Double distilled water
OD	Optical density
GC-MS	Gas chromatography-mass spectroscopy
TOC	Total organic carbon
IF	Induction Factor
MIF	Material induction factor
CIF	Control induction factor
LCB	Low-content brand
MCB	Medium-content brand
HCB	High-content brand
RT	Retention time
AUP	Area under the peak
PAH	Poly-aromatic hydrocarbons
ROS	Reactive oxygen species
