# Supplementary material for: Estimating Toxicity Putative Mechanisms from Smoking Residual Substances Using a Whole-Cell Bioreporter System
Source: Biosensors (Basel). 2025 Nov 3;15(11):733. doi: 10.3390/bios15110733 (PMC12650696; doi:10.3390/bios15110733)
Supplement: Supplementary file 1 [file biosensors-15-00733-s001.zip › biosensors-3762584-supplementary.pdf]

Supplementary:

In this study, cigarette smoke fractions were analyzed and tested for their toxicity using a bioluminescent bacteria array. An artificial smoking system that was developed in our lab was used to produce three cigarette smoke fractions from three different cigarette brands that differed in their tar and nicotine contents. Each fraction underwent GC-MS analysis, which enabled the identification of the compounds contained in the cigarette smoke and demonstrated the differences between the different cigarette brands. Testing of both the fractions and the pure compounds, alone or in mixtures, with the DPD2511 bacterial strain showed that they were significant triggers of oxidative stress, which was indicated by increased luminescence.

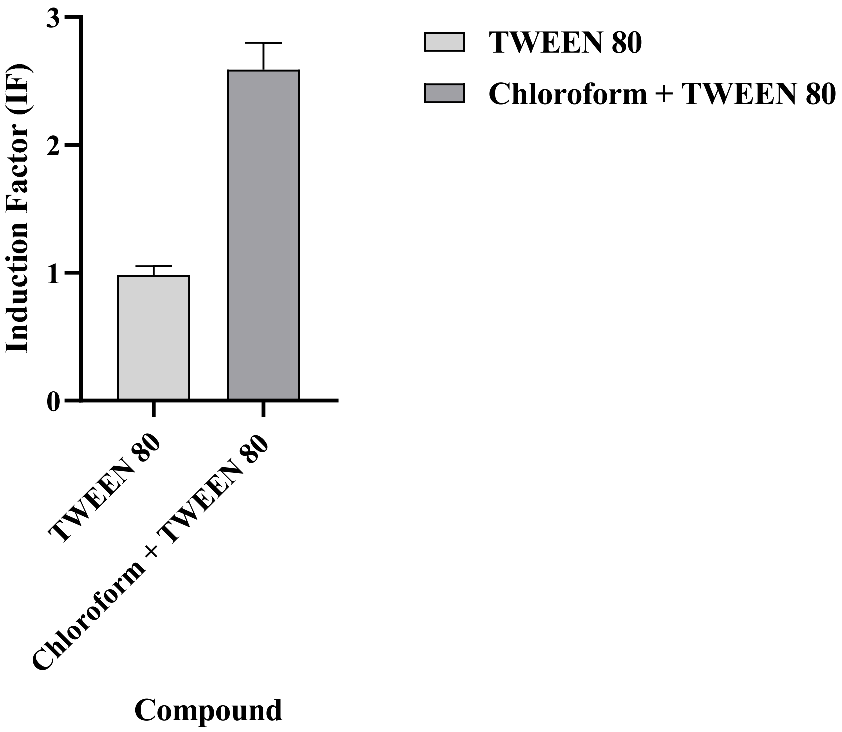

Figure S1. Hydrophobics solutions' additional compounds as oxidative stressors using DPD2511 bioreporter.

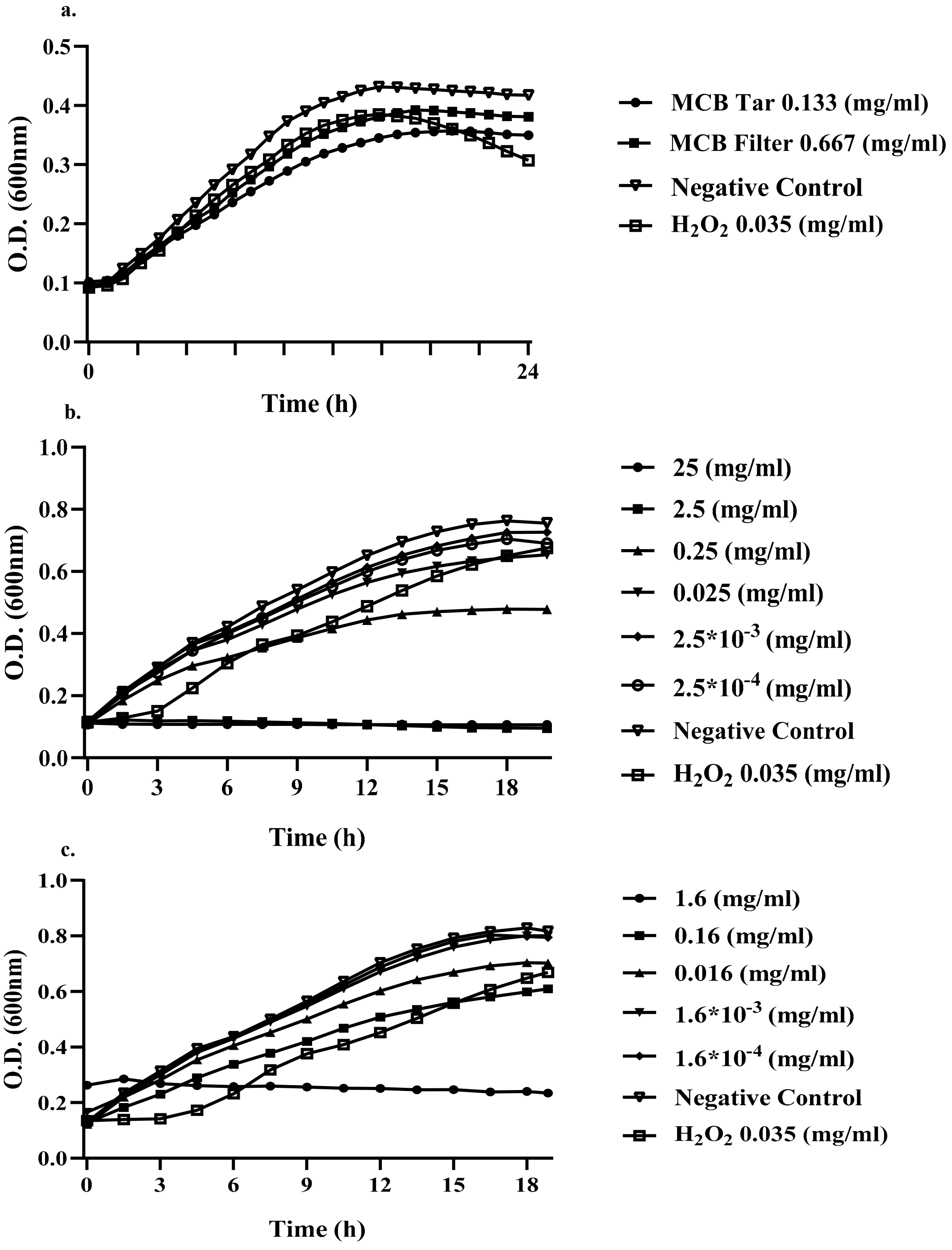

Figure S2. Kinetic analysis of the DPD2511 strain while exposed to cigarette fractions and single compounds. **(a)** Kinetic analysis of DPD2511 for both the Tar and Filter fractions from the medium content brand (MCB). **(b)** Kinetic analysis of DPD2511 for serial dilution concentrations of nicotine. **(c)** Kinetic analysis of DPD2511 for serial dilution concentrations of triacetin.
